# From Ordinary to Extraordinary: The Crucial Role of Common Species in Desert Plant Community Stability with Arbuscular Mycorrhizal (AM) Fungi Under Increased Precipitation

**DOI:** 10.3390/plants14071099

**Published:** 2025-04-02

**Authors:** Zhanquan Ji, Qianqian Dong, Rong Yang, Wenhao Qin, Yi Peng, Yangyang Jia

**Affiliations:** 1 College of Ecology and Environment, Xinjiang University, Urumqi 830046, China; 107552301776@stu.xju.edu.cn (Z.J.); 107552301742@stu.xju.edu.cn (Q.D.); 18199305490@163.com (R.Y.); 107552201244@stu.xju.edu.cn (W.Q.); 2 Key Laboratory of Oasis Ecology, Xinjiang University, Urumqi 830046, China; 3 College of Resources and Environment, Xinjiang Agricultural University, Urumqi 830052, China; pengyi0914@126.com

**Keywords:** Central Asian deserts, increased precipitation, arbuscular mycorrhizal fungi, temporal stability, common species stability, subordinate insurance hypothesis

## Abstract

Climate change is altering precipitation patterns in Central Asia’s arid zones, destabilizing desert ecosystems. Arbuscular mycorrhizal (AM) fungi, key soil microorganisms forming symbiosis with most plants, critically maintain ecosystem stability, yet their mechanisms in regulating individual plant species to sustain community stability remain unclear. We conducted a 5-year in situ experiment in the Gurbantunggut Desert, testing how AM fungi influence desert plant community stability under increased precipitation. Using a randomized block design with three treatments—control (CK), increased precipitation (W), and precipitation with Benomyl fungicide (BW)—we monitored plant community dynamics. We discovered that both increased precipitation and AM fungi altered plant community structure without affecting diversity. Precipitation boosted aboveground net primary productivity (ANPP) and density, enhancing community stability via dominant species (e.g., *Meniocus linifolius*), supporting the mass ratio hypothesis. AM fungi further stabilized the community by increasing ANPP and enhancing the common species stability under increased precipitation, while the contribution of rare species was also non-negligible, aligning with the subordinate insurance hypothesis. Overall, our study elucidates how increased precipitation and AM fungi regulate plant community stability at the species level. Specifically, it overcomes key gaps by revealing AM fungi’s pivotal role in stabilizing communities through sustaining common species stability.

## 1. Introduction

Within the context of accelerating global climate change, the frequency and intensity of extreme weather events have exhibited unprecedented growth trends; for example, precipitation has shown a fluctuating increasing trend [1,2,3,4,5]. Much of the earth is undergoing alterations in precipitation regimes, which poses significant impacts on the biomass, diversity, and ecosystem functions of terrestrial ecosystems globally [5,6,7]. Desert ecosystems are particularly vulnerable to precipitation variability due to water being the primary limiting factor, making them highly sensitive to changes in precipitation [8,9,10]. Currently, many temperate and mid-latitude deserts are experiencing rising precipitation, and similar trends have been observed in some Chinese deserts [4,11]. Existing studies have shown that increased precipitation can significantly impact the stability of these systems [12,13]. In desert ecosystems, soil microorganisms serve as the principal drivers, playing a pivotal role in soil biogeochemical processes and being instrumental in maintaining diverse ecosystem functioning [14,15]. Exploring the microbial interactions that control soil processes and how they promote the biodiversity-ecosystem functioning relationship in arid regions is crucial [16].

Arbuscular mycorrhizal (AM) fungi, widely distributed and highly diverse in desert ecosystems, play a significant role in their functioning [17,18]. AM fungi can form symbioses with over 90% of desert plant species, a proportion notably higher than the 80% observed in grassland ecosystems, making them a pivotal mediator in the response of desert ecosystems to climate change [19,20]. In return for carbohydrates supplied by the host plants, AM fungi confer numerous advantages, including enhanced nutrient uptake, mitigation of water stress, and augmented competitive abilities, among others [18,21,22]. This mutualistic relationship fosters species diversity and structural complexity within plant communities, thereby enhancing the overall stability and productivity of ecosystems [22,23]. Accumulated evidence has demonstrated that the absence of AM fungi can engender a cascade of deleterious consequences, leading to disruptions in plant nutrient cycling, impairing plant growth, and diminishing both productivity and stress resilience [12,24]. Ultimately, the stability of plant communities is compromised due to the disruption of the intricate network of interactions facilitated by AM fungi [25,26].

Stability is a fundamental property of ecosystems, and temporal stability represents the most commonly quantified dimension of this property in empirical research [27,28]. It can be assessed using the M. Godron stability index, which is a popular mathematical ecology approach internationally for measuring community stability [29,30]. It is relatively convenient and does not cause damage to the sampling plots. Furthermore, it has high credibility in reflecting the development and change trends of the community [31,32]. Understanding the impacts of different plant functional groups on plant community stability is a current focus in ecological research [33,34]. The mass ratio hypothesis posits that the immediate effects of plant species on ecosystem functions are proportional to their contributions to primary production. These effects are predominantly determined by the traits and functional diversity of dominant species while being relatively insensitive to the richness of subordinate and transient species [35]. Dominant species occupy primary ecological niches, and when water availability increases, they respond first due to their strong competitive ability, thereby determining the changes in the plant community [35,36,37]. However, the subordinate insurance hypothesis posits that subordinate plant species play a crucial yet underestimated role in maintaining ecosystem stability and function. Particularly under environmental perturbations, such as increased precipitation, these subordinate species can enhance the system’s resilience and compensate for potential losses of dominant species [38,39]. AM fungi can modulate interspecific competition, enhance the competitive ability of subordinate species, promote their growth, and subsequently contribute to the maintenance of community stability [23,25,40]. In recent years, a considerable volume of research has focused on the impact of increased precipitation and AM fungi on the stability of desert ecosystems [12,13,25]. Recent research has found that under increased precipitation and N deposition, AM fungi supported the stability of subordinate species, thereby maintaining the stability of the plant community [25]. However, the specifics at the species level remain unclear, particularly in extreme environments such as desert ecosystems where such research is notably scarce [12,34,41]. Addressing this knowledge gap is essential for effective ecological management and necessitates further in-depth investigation [16]. Given the unique habitat conditions and fragility of desert ecosystems, they serve as an ideal setting to test both hypotheses [42,43]. Therefore, this study aims to investigate how plant functional groups and individual plant species regulate the temporal stability of the desert plant community under increased precipitation, with a focus on the stabilizing role of AM fungi in buffering community dynamics.

Desert ecosystems constitute a crucial component of terrestrial ecosystems, with over 90% of the world’s temperate desert ecosystems located in the arid regions of Central Asia [44,45]. At present, Central Asian deserts are subject to intensified climate change, with notable alterations in precipitation patterns, especially a gradual overall increase in rainfall [11,46,47]. Moreover, due to the inherent fragility of this region’s environment and its low productive capacity, it exhibits a heightened sensitivity to precipitation change [4,5]. The Gurbantunggut Desert in China, being a significant component of the arid regions in Central Asia, serves as an ideal site for investigating the dynamic changes in desert ecosystems in the context of increased precipitation [12,48]. In this study, we conducted in situ experiments in the Gurbantunggut Desert of Xinjiang, China, with the following three treatments: control (CK), increased precipitation (W), and increased precipitation combined with AM fungi suppression (BW). Based on these experimental setups, we assessed the stability of the plant community, functional groups, and individual species. Furthermore, to uncover the underlying mechanisms through which increased precipitation and AM fungi affect community stability, we employed random forest (RF) models and structural equation modeling (SEM). Through these approaches, our primary objective is to address the following question: which functional groups and plant species contribute to enhancing the plant community stability under increased precipitation conditions with AM fungi? We hypothesized that (a) increased precipitation elevates plant community stability, while the absence of AM fungi diminishes it; (b) increased precipitation increases plant community stability through altering the dominant species stability; and (c) AM fungi increase plant community stability through altering the common species stability.

## 2. Results

### 2.1. Responses of Plant Community to Increased Precipitation and Suppression of AM Fungi

**Figure 1:**
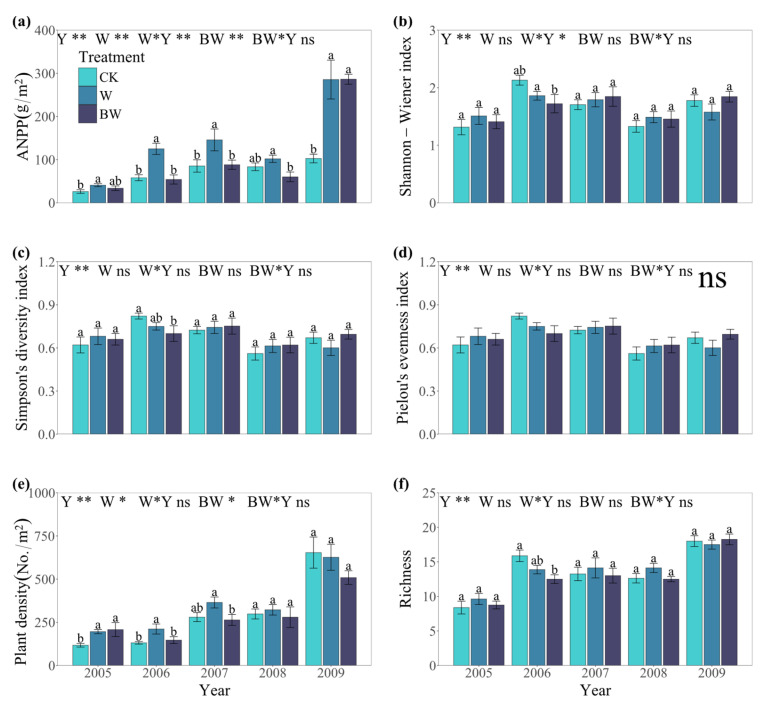
The ANPP (above-ground net primary productivity) (**a**), Shannon–Wiener index (**b**), Simpson’s diversity index (**c**), Pielou’s evenness index (**d**), plant density (**e**), and species richness (**f**) of the plant community under increased precipitation (W) and suppression of AM fungi (BW) during 2005–2009. Different bars indicate the mean value ± SE for the treatment. * and ** indicate statistical significance at *p* ≤ 0.05 and *p* ≤ 0.01, respectively. Different lowercase letters indicate significant differences among snow manipulation levels under major treatments (*p* < 0.05, Fisher’s LSD test). And ns indicate no significant differences. Y, year; CK, control; W, only water addition; BW, Benomyl with water. Same as the following.

Increased precipitation significantly enhanced the ANPP and plant density of the plant community but showed no significant alteration in Simpson’s diversity index, Pielou’s evenness index, or species richness (Figure 1 and Table S1). Suppression of AM fungi under increased precipitation significantly reduced the ANPP and plant density of the plant community without significantly affecting other community indicators (Figure 1, Table S1). Interannual differences for all community indicators were significant (Figure 1, Table S1). Over the five years, the highest values of ANPP, plant density, and species richness were recorded in 2009, followed by 2007, with lower values observed in the remaining years, corresponding to the respective precipitation conditions (Figure 1 and Figure S1b). The effects of increased precipitation on the plant community were more pronounced in 2006 compared to other years, resulting in greater increases in ANPP and plant density (Figure 1). Similarly, in 2006, the effects of suppression of AM fungi on the plant community were the most significant, leading to a substantial decrease in ANPP and plant density, highlighting the role of AM fungi in the plant community (Figure 1). Based on the results, it can be observed that the effect of increased precipitation is more pronounced in drought years, while AM fungi play a crucial role in resisting stress conditions.

**Figure 2:**
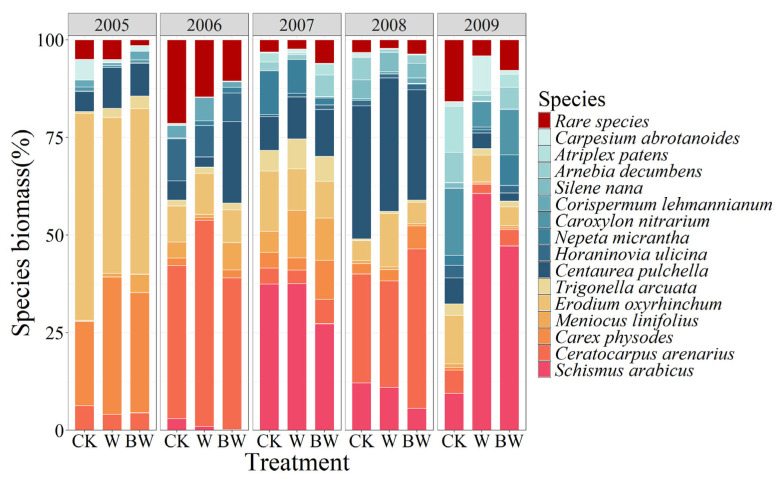
The relative biomass of each species and rare species overall within the plant community under increased precipitation (W) and suppression of AM fungi (BW) during 2005–2009. Orange represents 6 dominant species, blue represents 9 common species, and red represents overall rare species.

At the functional group level, increased precipitation elevated the biomass of dominant species but decreased the biomass of common species and rare species. The suppression of AM fungi under increased precipitation led to a reduction in the biomass of dominant species and an increase in the biomass of common and rare species (Figure S2 and Table S2). At the species level, increased precipitation significantly boosted the growth of several dominant species, including *Schismus arabicus*, *Ceratocarpus arenarius*, *Carex physodes*, *Meniocus linifolius*, and *Trigonella arcuata*, as well as some common species such as *Centaurea pulchella*, *Corispermum lehmannianum*, and *Carpesium abrotanoides*, but reduced the biomass of the remaining species (Figure 2 and Table S3). The suppression of AM fungi under increased precipitation reduced the biomass of dominant species such as *Schismus arabicus*, *Erodium oxyrhinchum*, and *Trigonella arcuata*, as well as common species including *Corispermum lehmannianum*, *Silene nana*, and *Carpesium abrotanoides*. Conversely, it increased the biomass of the remaining species (Figure 2 and Table S3).

The NMDS results demonstrated significant changes in the plant community structure under increased precipitation and AM fungi suppression, suggesting that the succession process of the plant community happened over the experimental investigation (Figure S3). On an interannual level, over five years, the dominant species transitioned from *Erodium oxyrhinchum* to *Ceratocarpus arenarius*, which was later supplanted by *Schismus arabicus*, achieving absolute dominance in the community (Figure 2).

### 2.2. The Influence Pattern of Increased Precipitation and AM Fungi on Plant Community Stability

**Figure 3:**
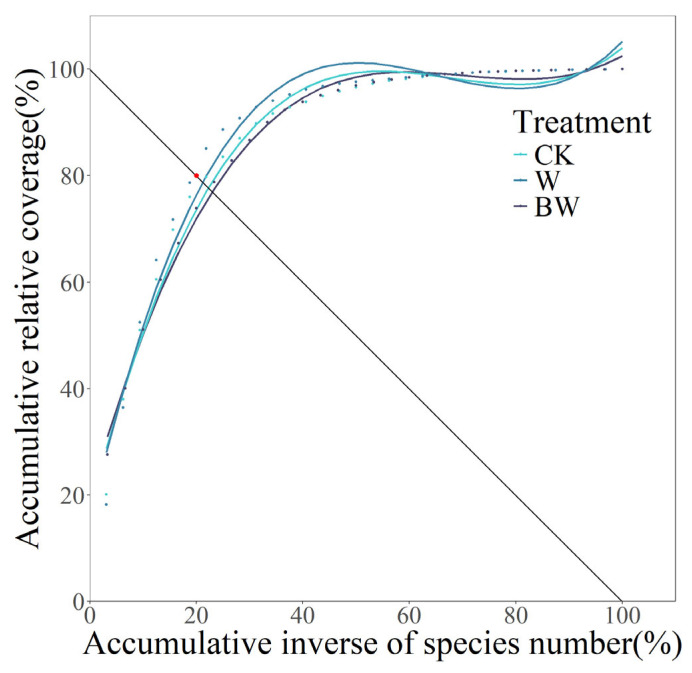
M. Godron stability fitted the curve of the plant community under increased precipitation (W) and suppression of AM fungi (BW) during 2005–2009. Points colored according to treatments represent the actual coordinates of each species under the corresponding treatment. The red dot on the straight line represents the reference point (20, 80).

The M. Godron stability index quantifies plant community stability by measuring the Euclidean distance between species coverage distributions and an ideal equilibrium state, with shorter distances indicating higher stability [29,49,50]. Utilizing the M. Godron stability index, we analyzed the plant community stability under three treatments (Figure 3). Each fitted curve showed good fit quality (R^2^ > 0.9, *p* < 0.01) (Table 1). The Euclidean distances from the intersection points of the curves under each treatment to the reference point (20, 80) were ranked in ascending order as W < CK < BW, indicating that the plant community under increased precipitation was the most stable, followed by the control, while the suppression of AM fungi led to the lowest plant community stability (Figure 3, Table 1). Under increased precipitation, the Euclidean distance was 1.4991, which was just 46.3% of the control, thus enhancing the plant community stability. However, under the suppression of AM fungi, the Euclidean distance was 4.5679, which was 3.05 times higher than that of the increased precipitation treatment, thereby reducing the plant community stability (Figure 3 and Table 1). Subsequently, we calculated the M. Godron stability index for three treatments over five years, with eight replicates each. The results were consistent with those mentioned above, indicating that increased precipitation led to the most stable plant community, whereas the suppression of AM fungi under increased precipitation significantly reduced community stability (Table S4). Using the ICV, we determined the stability of functional groups and individual species. For dominant and common species overall, both increased precipitation and AM fungi suppression reduced their stability, but the stability of individual species varied significantly (Table S5). For rare species, increased precipitation enhanced their stability, whereas suppression of AM fungi decreased their stability (Table S5).

**Figure 4:**
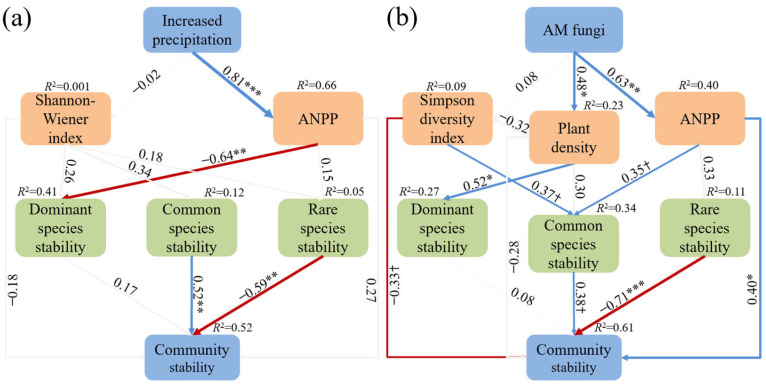
Structural equation modeling results of the effect of increased precipitation (**a**) (*p* = 0.928; RMSEA = 0.000; GFI = 0.951; AIC = 262.275; df = 10) and AM fungi (**b**) (*p* = 0.063; RMSEA = 0.203; GFI = 0.766; AIC = 313.860; df = 13) on plant community stability. Blue and red arrows denote significant positive and negative associations, respectively, while grey arrows indicate non-significant correlations. The arrow width is proportional to the strength of the relationship. Significance level: *** *p* < 0.001, ** *p* < 0.01, * *p* < 0.05 and † *p* < 0.1. Values along the arrows are standardized path coefficients, which represent relationships between variables. The R^2^ values, representing the proportion of variance explained, are presented along with the response variables in each analysis.

For increased precipitation, SEM collectively explained 52% of the variance in community stability (Figure 4a). It was ANPP, not the Shannon–Wiener index, which was altered by increased precipitation and subsequently influenced plant community stability. The common species stability was positive, but the rare species stability was negatively correlated with plant community stability. Notably, there were no significant relationships between the dominant species stability and plant community stability (Figure 4a). For AM fungi treatment, SEM collectively explained 61% of the variance in community stability (Figure 4b). AM fungi increased plant community stability by enhancing ANPP. Furthermore, AM fungi indirectly improved the common species stability through ANPP. The common species stability was positively correlated with plant community stability, thereby further reinforcing it (Figure 4b). The increase in plant density significantly enhanced the dominant species stability, but the contribution of dominant species stability to community stability was limited (Figure 4b). AM fungi did not affect Simpson’s diversity index, but Simpson’s diversity index was negatively correlated with community stability, and this index was positively correlated with the common species stability (Figure 4b). We also observed a significant negative correlation between the rare species stability and plant community stability (Figure 4b).

**Figure 5:**
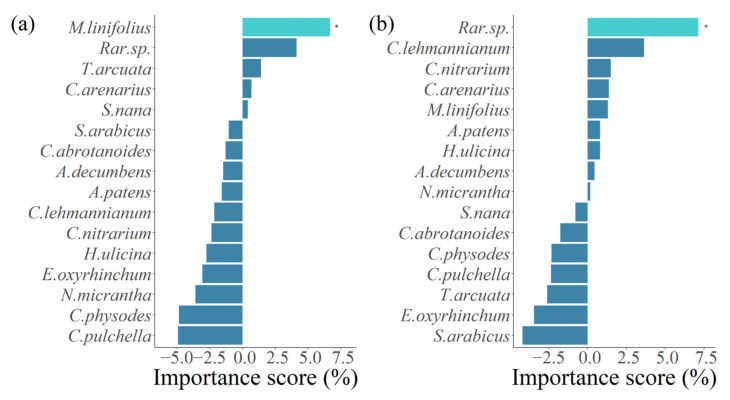
Random forests (RFs) analysis quantified the relative importance of individual species stability and rare species stability in determining plant community stability under increased precipitation (**a**) and AM fungi presence (**b**) (* *p*  <  0.05). Light blue indicates significant contributions, while dark blue denotes non-significant contributions.

Furthermore, RF analysis indicated that under increased precipitation, 5 species contribute positively to community stability, cumulatively accounting for 13.29% (Figure 5a and Table S6). Its dominant species, *Meniocus linifolius*, contributed the highest to plant community stability (6.68%), and this contribution was significant (Figure 5a and Table S6). The contributions of the remaining species to plant community stability were not significant. Among these species, *Meniocus linifolius*, *Trigonella arcuata*, and *Ceratocarpus arenarius* were dominant species, together contributing 8.77%, while *Silene nana* was a common species, contributing 0.4%. The contribution of rare species was only 4.12% (Figure 5a and Table S6). This indicates that under increased precipitation conditions, the dominant species stability contributes more to community stability. For AM fungi treatment, common species as a whole had the highest contribution to plant community stability at 7.34%. The contribution rate of rare species was 7.14% (Figure 5b and Table S6). Among these species, *Corispermum lehmannianum*, *Caroxylon nitrarium*, *Atriplex patens*, *Horaninovia ulicina*, *Arnebia decumbens*, and *Nepeta micrantha* are common species, while *Ceratocarpus arenarius* and *Meniocus linifolius* are dominant species, with a contribution rate of 2.68% (Figure 5b and Table S6). This demonstrated that with AM fungi, the contribution of common species to community stability is relatively large.

## 3. Discussion

Climate change threatens ecosystem functions and services, and the specific contributions of species to community stability under increased precipitation and AM fungi presence are still largely unknown [1,12,51]. We conducted a five-year in situ experiment in the Gurbantunggut desert to reveal the effects of increased precipitation and AM fungi on desert plant community stability at the species level. We found that increased precipitation enhances plant community stability through the dominant species stability. While common species begin to play more important roles than dominant species in maintaining plant community stability in the presence of AM fungi. These results fill the knowledge gap in understanding the effects of AM fungi on plant community stability at the species level in desert ecosystems.

### 3.1. Underlying Mechanisms of Increased Precipitation and AM Fungi on Plant Community Stability

In the present study, we found that increased precipitation significantly increased desert plant community stability, which is in line with previous studies [52,53,54]. Moreover, our results revealed that increased precipitation enhanced community stability by increasing the dominant species stability; this finding was consistent with previous research, which was conducted in temperate grasslands and tallgrass prairie, validating our previous hypothesis [55,56,57,58]. The temporal stability of a plant community is largely governed by its dominant species, which contributes to community stability via the “selection effect” [59,60,61]. Dominant species possess higher biomass, stronger survival capabilities, and greater adaptability. Due to their higher competitiveness or unique functional traits, they play a pivotal role in resource acquisition and utilization, thereby exerting a significant influence on the overall function of the community [59,62,63]. Among the dominant species, *Meniocus linifolius* showed the highest contributions to maintaining plant community stability. This can be attributed to several factors as follows: first, as an ephemeral plant, *Meniocus linifolius* has a high dependency on water with higher nitrogen and phosphorus reabsorption rates than those of other ephemeral plants [64]. Increased precipitation improves soil moisture and nutrient availability, thereby enhancing its survival rate, plant height, branching, and ultimately, biomass [64,65,66]. Second, its specialized life history strategy and the shortest lifespan minimize growth costs [65]. Additionally, its high phenotypic plasticity allows for leaf morphological adaptations to desert conditions, facilitating efficient resource utilization and energy accumulation [65,67]. Dominant species play a significant role in promoting community stability when precipitation increases, validating the mass ratio hypothesis and demonstrating their decisive role in the community [35,36,37].

Notably, rare species also showed significant contributions to the plant community stability, yet their low biomass and high species richness resulted in weak resistance to environmental disturbances and high variability. Consequently, their contribution to community stability may be transient [68,69]. The contribution of common species is low, possibly because the biomass and stability of common species individuals do not change significantly when precipitation increases, resulting in no observed contribution of common species stability to community stability [70,71,72]. Our study also showed that increased precipitation posed limited effects on species diversity, consistent with previous research [13,36]. However, we did not observe a positive correlation between diversity and stability, which is different from the earlier studies [73,74]. This suggests that in our study, the impact of precipitation mainly manifests in productivity rather than changes in species diversity. However, this does not exclude other factors such as soil quality, climate conditions, and biotic disturbances that may affect the relationship between species diversity and stability [51,75,76].

AM fungi play indispensable roles in maintaining plant community stability [12,25,77]. In our research, we found that AM fungi increased the ANPP and common species stability and subsequently enhanced the plant community stability. This indicates that AM fungi significantly boost ANPP, which aligns with previous findings in desert ecosystems and indoor simulation experiments [25,78]. One previous study underscores that AM fungi can mitigate the adverse impacts of global changes on plant growth and foster species coexistence [79]. Their presence enhances community stability, whereas the absence of AM fungi leads to a marked reduction in plant community stability—a conclusion that aligns with our research [12,77,79]. These results highlight the critical role of AM fungi in plant growth through their mutualistic symbiosis, which increases overall community biomass and, in turn, community stability [25,77]. AM fungi did not significantly influence Simpson’s diversity index; however, this index was found to be negatively correlated with community stability, which is consistent with previous research findings [75,80]. When Simpson’s diversity index approaches 0, indicating a more even distribution of relative species abundances and higher diversity levels, its trend is inversely related to community stability, resulting in a negative correlation [81].

In the presence of AM fungi, it is the common species stability that enhances plant community stability. At the species level, similar results were obtained as those at the functional group level. This finding indicates that AM fungi predominantly increase the common species stability, reflecting the subordinate insurance hypothesis [38,39]. Three factors might explain the high contribution of common species to community stability with AM fungi as follows: first, in a community, AM fungi serve as stabilizers. Different host-specific mycorrhizal fungi with diverse functional roles can enhance the allocation of nutrient niches, facilitating the coexistence of various plant species [82]. Consequently, AM fungi can mitigate the competitive pressure exerted by dominant species on common species, thereby helping maintain higher growth rates and biomass in common species through their nutrient redistribution mechanisms, particularly under resource-limited conditions [23,25,40]. Second, the distinct functional traits of these species confer higher adaptability. For example, *Corispermum lehmannianum* demonstrates strong adaptability to environmental changes; its seeds, after drying due to a lack of water post-germination, can continue to grow young roots upon rehydration [83,84]. *Caroxylon nitrarium* exhibits high tolerance to both saline and arid conditions, indicating strong environmental adaptability [85]. Third, despite AM fungi enhancing the dominant species stability, their contribution to community stability may remain limited, as the functional diversity and stress-adaptive strategies of common species are more critical in buffering environmental variability [38,39]. Rare species are often considered less impactful due to their low biomass and high functional redundancy [68,69,86]. However, in this study, the majority of rare plant species are mycorrhizal-dependent [19,87]. When AM fungi were present, these rare species significantly enhanced community stability, as AM fungi facilitated their access to soil water and nutrients, allowing persistence despite competition with dominant species [88,89]. Although rare species have limited individual biomass, their collective contributions to stability become vital under the AM fungal network [88,90]. The contributions of both common and rare species to community stability have been historically overlooked. However, emerging evidence demonstrates that variations in the common species stability make the greatest contribution to the biomass stability of plant communities [91]. A deeper understanding of the precise mechanisms by which AM fungi regulate community stability through common species requires more specific and targeted scientific investigations.

### 3.2. The Impacts of Increased Precipitation and AM Fungi on Plant Community Structure

Numerous studies in semi-arid sandy lands, Central Asian desert ecosystems, and temperate desert steppes have shown that increased precipitation significantly enhances the ANPP and plant density of desert plant communities, and our results further confirm these findings [92,93,94,95]. This underscores the critical role of precipitation in mediating the responsiveness of plant community characteristics to environmental change [96,97]. Availability of soil water and interspecific competition are identified as pivotal factors driving shifts in plant community composition [13,98]. Dominant species, characterized by their high initial biomass, exhibit superior competitive capabilities compared to the other functional groups and display greater resilience to environmental changes. This enables them to respond more effectively to increased precipitation, resulting in a notable increase in ANPP [52,92,94]. Furthermore, the increases in soil moisture intensify interspecific competition for other resources, placing other species at a competitive disadvantage. This, in turn, results in a decline of common and rare species biomass. These species already have lower competitive ability and colonization rates, and their growth may be further suppressed due to narrow ecological niches [13,99]. At the species level, individual species also exhibit similar response patterns to those of functional groups. Interestingly, during the five-year study period, we observed a succession of dominant species in the plant community, which may be the result of community succession, as confirmed by our NMDS results [100].

AM fungi play pivotal roles in various ecosystems, exhibiting tight associations with other soil microorganisms and exerting significant influence within microbial communities [101,102]. They can enhance plant adaptation to environmental changes and promote plant growth by optimizing resource allocation [103]. Under increased precipitation, AM fungi result in a significant increase in both ANPP and plant density, which aligns with extensive previous research on AM fungi [12,25,104]. AM fungi may alleviate the negative impacts of environmental stress on plant growth in several ways [103]. First, AM fungi can alter soil structure and soil water status [105]. Second, AM fungi can access nutrients and water from the soil through an extensive hyphal network and transport them to the host plants [106]. Additionally, AM fungi can enhance the availability of soil nutrients, thereby promoting nutrient uptake by plants [107]. Therefore, all plant species biomass was increased with AM fungi, but the biomass proportion remains dominated by the dominant species, followed by the common species. Meanwhile, rare species, due to being less affected, show minimal changes in ANPP. This underscores the indispensable role of AM fungi in modulating plant productivity and community composition and their paramount importance in maintaining the stability of the plant community [79,101,102].

While this study reveals the critical mechanism by which AM fungi enhance desert community stability through regulating common species, the following three limitations persist: (1) the five-year observation period captured only short-term response patterns of the plant community; (2) failure to resolve functional heterogeneity among AM fungal strains; and (3) lack of data on interactions between mycorrhizal networks and soil micro-food webs (e.g., nematode-hyphal predation interactions). Future studies should employ cross-aridity gradient network experiments integrating isotopic tracing (e.g., ^15^N/^33^P labeling for resource allocation tracking) and multi-omics techniques to quantify plant-soil feedback thresholds under long-term climate scenarios while constructing tripartite “fungi-plant-animal” interaction models. These advances will lay a solid foundation for ecological restoration and stability maintenance in arid regions.

## 4. Materials and Methods

### 4.1. Study Site

**Figure 6:**
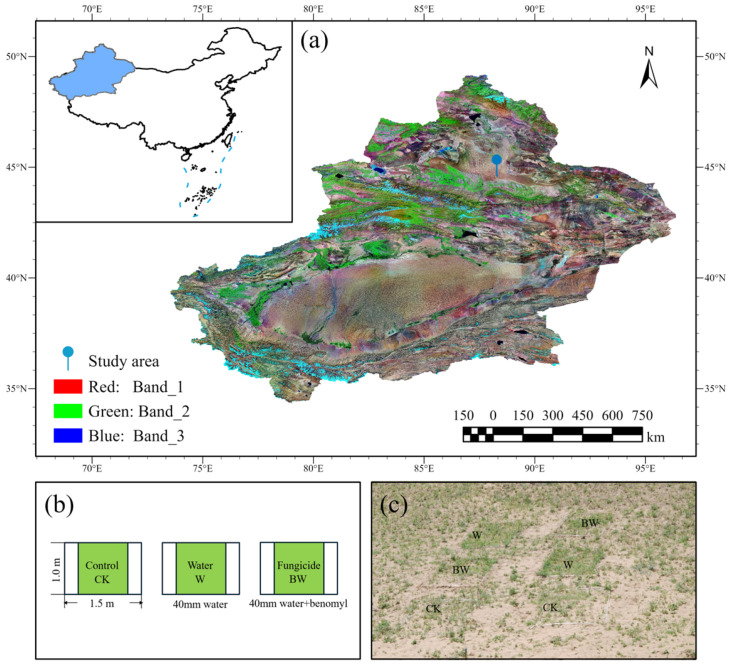
Study area location ((**a**); map created by authors), experimental design schematic ((**b**); designed by T. Zhang), and field implementation photographs ((**c**); photo credit: T. Zhang) [114]. The map in panel (**a**) was first generated for this study using Landsat-7 ETM+ imagery (USGS). CK, control; W, only water addition; BW, Benomyl with water.

The study site is located at the southern edge of the Gurbantunggut Desert (latitude 88°28′ E, longitude 44°54′ N) (Figure 6a). The Gurbantunggut Desert, centrally located in the Junggar Basin of Xinjiang, Central Asia, is the largest fixed and semi-fixed desert in China. It spans elevations ranging from 249 to 868 m above sea level and covers a total area of approximately 48,800 km^2^ [108]. This area has a typical continental arid, temperate climate, with hot summers and cold winters. The mean annual temperature is around 7.19 °C, with an annual precipitation range of 100–200 mm, primarily distributed across spring and autumn seasons, and the annual evapotranspiration exceeds 2000 mm [109,110]. The annual precipitation during the experiment did not exceed 250 mm (Figure S1a). Soils are gray desert soils (Chinese classification) with aeolian sands on the surface (0–100 cm) [111]. Snowmelt and spring precipitation serve as the primary sources of moisture for vegetation in this desert, accounting for over 65% of the annual rainfall [112]. This abundance of early spring water provides favorable conditions for the growth of ephemeral plants [84,113]. Prominent among the herbaceous vegetation are species such as *Schismus arabicus*, *Ceratocarpus arenarius*, *Carex physodes*, *Meniocus linifolius*, *Erodium oxyrhinchum*, and *Trigonella arcuate* [12] (Table S7).

### 4.2. Experimental Design

The experiment was initiated in the early spring of 25 March 2005 and concluded in 2009, utilizing a randomized block design that included three treatments as follows: control (CK, no additional watering or fungicide application), increased precipitation (W), and increased precipitation coupled with Benomyl fungicide (BW) (Figure 6b,c). To prevent grazing and trampling by animals, the experimental plots were enclosed with iron fences in early March 2005, before the germination of ephemeral desert plants. Based on previous research indicating a projected increase in precipitation of 40 mm over the next 50 to 100 years, we implemented an increment of 40 mm to simulate future precipitation patterns [115,116]. The study area was divided into five sections, and one section was surveyed and harvested each year. Each section contained 24 quadrats (1 × 1.5 m^2^ in size), with eight replicates per treatment. Quadrats were spaced 2 m apart from adjacent ones. The central 1 × 1 m^2^ portion of each quadrat served as the sampling plot, surrounded by a buffer zone to minimize edge effects (Figure 6b,c).

The experiment was conducted annually from 25 March to 25 May, spanning the period from plant emergence to biomass peak. Precipitation was augmented by adding 15 L of water to each quadrat every two weeks, equivalent to an extra 10 mm of precipitation, with a total of four applications during the growing season. For the treatment aimed at suppressing the activity of AM fungi (BW), we employed the method of Benomyl soil drenches. Benomyl was applied at a consistent water volume (9 g of active ingredient in 15 L of water) every two weeks, given that Benomyl cannot be applied alone and requires dilution in water [12,77,117]. Benomyl, a systemic fungicide, effectively disrupts AM fungal hyphal growth and root colonization by specifically targeting microtubule assembly in AM fungi [117,118,119]. Despite sharing the original dataset with Jia et al. (2022), this study adopts distinct research perspectives [12]. Notably, Jia’s study demonstrated that Benomyl treatment significantly reduces the hyphal density and spore density of AM fungi [12]. Furthermore, Benomyl has been shown to have minimal edge effects and negligible impacts on non-target fungi and soil microorganisms [117,120].

During the growing season, the study area received an average annual precipitation of 69.7 mm and an average temperature of 10.2 °C (Figure S1b). Based on the average precipitation during the growing season, 2009 was categorized as a wet year, whereas 2005, 2006, and 2008 were classified as dry years, and 2007 as a normal year (Figure S1b) [121].

### 4.3. Sampling

When the plant community ANPP reached its peak, we measured the plant coverage and harvested the shoots of the plants in each quadrat. Each plant was taxonomically identified, and subsequently, plant density and species richness were calculated. Following the harvest, plant samples were sorted by species and subsequently subjected to enzyme deactivation at 105 °C for 0.5 h and then oven-dried at 65 °C for 48 h. Soil water content was measured using the gravimetric method [122]. Specific details followed the methods described by Jia et al. (2022) [12].

In the community, plants are categorized into the following three functional groups based on their relative abundance as follows: dominant species, common species, and rare species. Dominant, common, and rare species were defined as having relative abundances greater than 5%, between 1% and 5%, and less than 1%, respectively [7,123]. The community consists of 39 species, including 6 dominant species, 9 common species, and 24 rare species, accounting for 65.14%, 27.71%, and 7.15% of the community biomass, respectively (Table S7).

### 4.4. Plant Community Diversity and Stability Analysis

To characterize the plant community diversity, we quantified the Shannon–Wiener index (1), Simpson’s diversity index (2), Pielou’s evenness index (3), and species richness (4) within the plant community [81,124,125], with calculations as follows:

(1) $$H=-\sum_{i=1}^{S}P_{i\;}\ln\left(P_{i\;}\right)$$

(2) $$D=1-\sum_{i=1}^{S}{P_{i}}^{2}$$

(3) $$J=\frac{H}{\ln\left(S\right)}$$

(4) R=S

where $P_{i}$ is the relative abundance of *i*th species within a quadrat ($P_{i}=N_{i}/N$, N is the total number of individuals in a quadrat; $N_{i}$ is the number of individuals of *i*th species in a quadrat); and *S* is the total number of species in a quadrat.

Community temporal stability: the modified M. Godron stability index was employed to evaluate community temporal stability [29,31,126]. The M. Godron stability index is more convenient compared to other methods and has high credibility in reflecting the development and change trends of the community [31,32]. Prior research has indicated that stability assessments based on coverage yield more compelling results; therefore, in our study, we used the same methods [127]. Additionally, to enhance the goodness-of-fit of fitted curves, a third-degree polynomial function was adopted. First, sort all plant species in the community in descending order of their coverage. Calculate the relative coverage of each species (the coverage of each species divided by the total coverage of all species), and then accumulate these values to obtain the cumulative relative coverage (%) for each species. Next, based on the above sorting, calculate the cumulative reciprocal of the number of species for each species (the current species sequence number divided by the total number of species, %). Plot a scatter diagram with the cumulative reciprocal of the number of species as the x-axis and the cumulative relative coverage as the y-axis, and establish a smooth curve model using the cubic equation $y={ax}^{3}+{bx}^{2}+cx+d$ [127]. Finally, plant community stability was obtained under the three treatments over these five years. The intersection point of the smooth curve with the straight line y=100−x in the first quadrant was determined, and a shorter Euclidean distance from this intersection to the point (20,80) along the straight line corresponded to higher stability of the plant community [29,127]. To meet the data requirements for both SEM and RF, we also calculated the community stability for the eight replicates of each of the three treatments over the five years (Table S4).

Functional groups and individual species stability: to assess the stability of individual species and three functional groups, the inverse coefficient of variation (ICV) was calculated as the ratio of the temporal mean biomass (μ) to its temporal standard deviation (σ) at the quadrant scale over the five years [128,129].

### 4.5. Statistical Analysis

First, a three-way repeated-measures analysis of variance (ANOVA) was performed to test the main and interactive effects of the year (Y), increased precipitation (W), and suppression of AM fungi (BW) on ANPP, plant density, Shannon–Wiener index, Simpson’s diversity index, Pielou’s evenness index, and species richness (Table S1). One-way ANOVA was employed to assess the impacts of increased precipitation and the suppression of AM fungi on the aforementioned community indices across different years. ANOVA was performed using IBM SPSS Statistics software (version 25.0; IBM Corporation, Armonk, NY, USA). Second, non-metric multidimensional scaling (NMDS) was employed to analyze the impact of increased precipitation and AM fungi on the plant community structure over five years.

Finally, SEM was employed to elucidate the potential pathways influencing plant community stability under increased precipitation and AM fungi presence. The fitness of the model was evaluated using the χ^2^ test, root-mean-square error of approximation (RMSEA), goodness-of-fit index (GFI), and Akaike information criterion (AIC) [130]. SEM was conducted using R software (version 4.4.1; R Foundation for Statistical Computing, Vienna, Austria) with the “lavaan” package. Furthermore, the RF model was utilized to quantify the contribution rates of dominant species, common species, and rare species to the temporal stability of the plant community under increased precipitation and AM fungi presence. RF was performed in R 4.4.1 with the “randomForest” package.

## 5. Conclusions

This study, through a five-year in situ experiment, revealed the regulatory mechanisms of AM fungi on plant community stability in the Gurbantunggut Desert under increased precipitation. The main findings are as follows:

(1) Dominant species drive community stability under increased precipitation. Increased precipitation enhanced ANPP and plant density and improved community stability. The dominant plant *Meniocus linifolius* was the primary contributor to community stability, supporting the mass ratio hypothesis. Dominant species dominated community dynamics under increased water availability due to their superior resource competitiveness and phenotypic plasticity.
(2) AM fungi stabilize plant communities by enhancing ANPP and common species stability. AM fungi significantly increased the ANPP and plant density. By enhancing ANPP and stabilizing common species, AM fungi maintained plant community stability. Furthermore, the overall contribution of rare species to community stability was non-negligible, thereby validating the subordinate insurance hypothesis. AM fungi alleviated competitive pressure from dominant species through nutrient redistribution and improved the environmental adaptability of subordinate species.
(3) ANPP, not diversity, mediates community stability across treatments. None of the treatments altered plant diversity, indicating that community stability was primarily driven by ANPP rather than diversity.

These findings provide the first species-level evidence that AM fungi maintain desert community stability by regulating the common species stability and highlight their role as stabilizers in arid ecosystems. This study fills a key knowledge gap in species-level plant-microbial interactions in arid ecosystems and provides a theoretical foundation for the adaptive management of desert ecosystems under climate change.

## Figures and Tables

**Table 1:** Temporal stability of the plant community under increased precipitation (W) and suppression of AM fungi (BW) during 2005–2009. CK, control; W, only water addition; BW, Benomyl with water.

Treatment	Fitted Curve	Correlation Coefficient (R^2^)	*p* Value	Intersection Coordinate	Euclidean Distance
CK	y = 0.0003x^3^ − 0.0602x^2^ + 3.9091x + 17.1632	0.9794	*p* < 0.01	(22.29, 77.71)	3.2385
W	y = 0.0004x^3^ − 0.0698x^2^ + 4.3200x + 15.1589	0.9682	*p* < 0.01	(21.06, 78.94)	1.4991
BW	y = 0.0002x^3^ − 0.0517x^2^ + 3.5506x + 19.6804	0.9948	*p* < 0.01	(23.23, 76.77)	4.5679

## Data Availability

Data are contained within this article and Supplementary Materials.

## Supplementary Materials

The following supporting information can be downloaded at: https://www.mdpi.com/article/10.3390/plants14071099/s1, Table S1: Results of three-way repeated-measures ANOVA: Effects of year (Y), increased precipitation (W), suppression of arbuscular mycorrhizal (AM) fungi (BW), and their interactions on above-ground net primary productivity (ANPP), plant diversity, and plant density; Table S2: The relative abundance of three functional groups within the plant community based on ANPP under increased precipitation (W) and suppression of AM fungi (BW) during 2005–2009; Table S3: The relative biomass of each species and rare species overall within the plant community under increased precipitation (W) and suppression of AM fungi (BW) during 2005–2009; Table S4: Temporal stability of plant community across three treatments (CK, W, BW) with eight replicates per treatment during 2005–2009; Table S5: The stability of three functional groups and all dominant and common species within the plant community under three treatments during 2005–2009; Table S6: Random forests (RF) analysis revealed the importance scores of individual species’ stability and overall rare species’ stability in predicting plant community stability under increased precipitation and AM fungi presence; Table S7: Latin names and relative abundance (RA) of dominant, common, and rare species in the desert plant community during 2005–2009 (39 species in total); Figure S1: Changes in total precipitation and mean air temperature at the experimental site for the whole year (a) and the growing season (March–May) (b) from 2005 to 2009; Figure S2: The relative abundance of three functional groups within the plant community based on ANPP under increased precipitation (W) and suppression of AM fungi (BW) during 2005–2009; Figure S3. NMDS analysis shows the impact of increased precipitation (W) and suppression of AM fungi (BW) on plant community structure from 2005 to 2009.
