# Supplementary material for: From Ordinary to Extraordinary: The Crucial Role of Common Species in Desert Plant Community Stability with Arbuscular Mycorrhizal (AM) Fungi Under Increased Precipitation
Source: Plants (Basel). 2025 Apr 2;14(7):1099. doi: 10.3390/plants14071099 (PMC11991153; doi:10.3390/plants14071099)
Supplement: Supplementary file 1 [file plants-14-01099-s001.zip › plants-3534091-supplementary.pdf]

plants

MDPI

Table S1. Results of three-way repeated-measures ANOVA: Effects of year (Y), increased precipitation (W), suppression of arbuscular mycorrhizal (AM) fungi (BW), and their interactions on above-ground net primary productivity (ANPP), plant diversity, and plant density.

	Source of variation				
	**Y(df=4)**	**W(df=1)**	**W*Y(df=4)**	**BW(df=1)**	**BW*Y(df=4)**
ANPP	**< 0.01**	**< 0.01**	**< 0.01**	**< 0.01**	0.227
Shannon-Wiener index	**< 0.01**	0.921	**0.035**	0.884	0.388
Simpson's diversity index	**< 0.01**	0.931	0.157	0.777	0.541
Pielou's evenness index	**< 0.01**	0.831	0.272	0.521	0.688
Plant density	**< 0.01**	**0.024**	0.347	**0.02**	0.536
Richness	**< 0.01**	0.604	0.076	0.051	0.423

Note: *p*-values in bold are significantly different ($p < 0.05$). Y, year; W, only water addition; BW, Benomyl with water.

Table S2. The relative abundance of three functional groups within the plant community based on ANPP under increased precipitation (W) and suppression of AM fungi (BW) during 2005-2009.

Functional groups	Treatment		
	CK	W	BW
Dominant species	58.7	70.47	66.26
Common species	31.54	23.81	27.79
Rare species	9.76	5.73	5.95

Table S3. The relative biomass of each species and rare species overall within the plant community under increased precipitation (W) and suppression of AM fungi (BW) during 2005-2009.

**Species**	**Treatment**		
	**CK**	**W**	**BW**
Schismus arabicus	12.41	22.03	16.05
Ceratocarpus arenarius	16.67	17.97	18.92
Carex physodes	6.18	8.48	9.82
Meniocus linifolius	2.29	2.96	4.73
Erodium oxyrhinchum	19.05	16.30	14.11
Trigonella arcuata	2.10	2.73	2.63
Centaurea pulchella	11.90	12.40	14.36
Horaninovia ulicina	3.17	2.16	2.36
Nepeta micrantha	2.84	2.12	2.26
Caroxylon nitrarium	3.72	1.42	2.54
Corispermum lehmannianum	1.07	1.45	1.03
Silene nana	1.23	1.03	0.76
Arnebia decumbens	3.08	0.72	2.65
Atriplex patens	2.82	0.36	1.22
Carpesium abrotanoides	1.71	2.15	0.61
Rare species	9.76	5.73	5.95

Table S4. Temporal stability of plant community across three treatments (CK, W, BW) with eight replicates per treatment during 2005-2009.

Treatment	Block	Fitted curve	R^2	*p*-value	Intersection coordinate	Euclidean distance	Reciprocal Euclidean distance
CK	1	$y=0.00022x^3-0.0482x^2+3.6021x+4.3603$	1.00	$p<0.01$	(27.89,72.11)	11.16	0.09
CK	2	$y=0.00013x^3-0.0309x^2+2.6956x+14.3458$	1.00	$p<0.01$	(29.58,70.42)	13.55	0.07
CK	3	$y=0.00011x^3-0.0289x^2+2.6939x+7.9863$	1.00	$p<0.01$	（31.91,68.09）	16.84	0.06
CK	4	$y=0.00023x^3-0.0509x^2+3.6907x+9.1854$	1.00	$p<0.01$	（25.69,74.31）	8.05	0.12
CK	5	$y=0.00022x^3-0.0498x^2+3.8378x-1.6914$	1.00	$p<0.01$	（26.87,73.13）	9.72	0.10
CK	6	$y=0.00021x^3-0.0479x^2+3.6534x+3.8350$	1.00	$p<0.01$	（27.52,72.48）	10.63	0.09
CK	7	$y=0.00019x^3-0.0437x^2+3.3981x+9.2355$	1.00	$p<0.01$	（27.05,72.95）	9.97	0.10
CK	8	$y=0.00017x^3-0.0385x^2+2.9624x+20.3923$	0.99	$p<0.01$	（25.84,74.16）	8.26	0.12
W	1	$y=0.00026x^3-0.0549x^2+3.7355x+13.3705$	0.99	$p<0.01$	（24.40,75.60）	6.22	0.16
W	2	$y=0.00022x^3-0.0480x^2+3.6076x+4.5466$	0.99	$p<0.01$	（27.69,72.31）	10.88	0.09
W	3	$y=0.00017x^3-0.0404x^2+3.3268x+2.1767$	1.00	$p<0.01$	（29.91,70.09）	14.01	0.07
W	4	$y=0.00017x^3-0.0419x^2+3.5092x-0.2784$	1.00	$p<0.01$	（29.24,70.76）	13.07	0.08
W	5	$y=0.00019x^3-0.0451x^2+3.6782x-1.8399$	1.00	$p<0.01$	（28.79,71.21）	12.43	0.08
W	6	$y=0.00024x^3-0.0519x^2+3.7750x+5.3242$	1.00	$p<0.01$	（26.55,73.45）	9.26	0.11
W	7	$y=0.00013x^3-0.0349x^2+3.1082x+5.7310$	1.00	$p<0.01$	（29.55,70.45）	13.51	0.07
W	8	$y=0.00025x^3-0.0537x^2+3.8575x+5.5933$	1.00	$p<0.01$	（26.01,73.99）	8.50	0.12
BW	1	$y=0.00011x^3-0.0284x^2+2.6583x+12.4088$	1.00	$p<0.01$	（30.19,69.81）	14.42	0.07
BW	2	$y=0.00013x^3-0.0334x^2+2.8529x+16.1877$	1.00	$p<0.01$	（27.68,72.32）	10.86	0.09
BW	3	$y=0.00021x^3-0.0472x^2+3.5759x+2.9994$	1.00	$p<0.01$	（28.53,71.47）	12.06	0.08
BW	4	$y=-0.00002x^3-0.0081x^2+1.8684x+10.5068$	1.00	$p<0.01$	（34.91,65.09）	21.09	0.05
BW	5	$y=0.00020x^3-0.0430x^2+3.1903x+16.2102$	0.99	$p<0.01$	（26.17,73.83）	8.73	0.11
BW	6	$y=0.00015x^3-0.0344x^2+2.8257x+16.1535$	1.00	$p<0.01$	（28.18,71.82）	11.57	0.09
BW	7	$y=0.00018x^3-0.0399x^2+3.0503x+16.2494$	0.99	$p<0.01$	（26.97,73.03）	9.86	0.10

BW	8	$y=0.00008x^3-0.0211x^2+2.0662x+26.2040$	1.00	$p<0.01$	（29.33,70.67）	13.19	0.08

Table S5. The stability of three functional groups and all dominant and common species within the plant community under three treatments during 2005-2009.

**Stability**		**CK**	**W**	**BW**
Functional groups stability	Dominant species	1.96	1.47	1.11
	Common species	1.23	1.22	0.83
	Rare species	0.90	0.92	0.78
Species stability	*M.linifolius*	0.76	0.92	0.94
	C.physodes	1.09	0.96	1.13
	E.oxyrhinchum	1.22	1.92	1.30
	T.arcuata	0.85	0.97	0.97
	C.arenarius	1.01	0.85	1.44
	S.arabicus	0.99	0.73	0.74
	C.pulchella	0.90	1.02	0.91
	C.lehmannianum	0.76	3.78	0.80
	C.abrotanoides	1.60	0.87	3.95
	C.nitrarium	0.54	0.46	0.44
	H.ulicina	1.08	0.95	0.94
	A.patens	0.35	0.22	0.69
	N.micrantha	2.03	1.96	3.22
	S.nana	0.30	0.61	0.24
	A.decumbens	0.60	1.38	1.47

Table S6. Random forests (RF) analysis revealed the importance scores of individual species' stability and overall rare species' stability in predicting plant community stability under increased precipitation and AM fungi presence.

Treatment	Species	Functional group	Importance score (%)	*p*-value
Increased precipitation	*Meniocus linifolius*	Dominant species	6.68	0.03
	Rare species	Rare species	4.12	0.13
	Trigonella arcuata	Dominant species	1.41	0.23
	Ceratocarpus arenarius	Dominant species	0.68	0.25
	Silene nana	Common species	0.40	0.31
	Schismus arabicus	Dominant species	-1.06	0.41
	Carpesium abrotanoides	Common species	-1.29	0.50
	Arnebia decumbens	Common species	-1.47	0.55
	Atriplex patens	Common species	-1.58	0.68
	Corispermum lehmannianum	Common species	-2.15	0.69
	Caroxylon nitrarium	Common species	-2.36	0.71
	Horaninovia ulicina	Common species	-2.74	0.86
	Erodium oxyrhinchum	Dominant species	-3.07	0.89
	Nepeta micrantha	Common species	-3.58	0.88
	Carex physodes	Dominant species	-4.85	0.98
	Centaurea pulchella	Common species	-4.92	1.00
AM fungi	Rare species	Rare species	7.14	0.05
	Corispermum lehmannianum	Common species	3.64	0.09
	Caroxylon nitrarium	Common species	1.49	0.15
	Ceratocarpus arenarius	Dominant species	1.37	0.19
	Meniocus linifolius	Dominant species	1.31	0.25
	Atriplex patens	Common species	0.81	0.29
	Horaninovia ulicina	Common species	0.80	0.34
	Arnebia decumbens	Common species	0.44	0.31
	Nepeta micrantha	Common species	0.16	0.33
	Silene nana	Common species	-0.78	0.44
	Carpesium abrotanoides	Common species	-1.75	0.73
	Carex physodes	Dominant species	-2.32	0.74
	Centaurea pulchella	Common species	-2.36	0.68
	Trigonella arcuata	Dominant species	-2.60	0.82
	Erodium oxyrhinchum	Dominant species	-3.46	0.90
	Schismus arabicus	Dominant species	-4.19	0.94

Table S7. Latin names and relative abundance (RA) of dominant, common, and rare species in the desert plant community during 2005-2009 (39 species in total).

Dominant species		Common species		Rare species	
**Latin name**	**RA(%)**	**Latin name**	**RA(%)**	**Latin name**	**RA(%)**
Schismus arabicus	29.65	*Centaurea pulchella*	3.22	*Nepeta pungens*	0.91
Ceratocarpus arenarius	19.53	*Horaninovia ulicina*	2.63	*Corispermum squarrosum*	0.78
Carex physodes	9.42	*Nepeta micrantha*	2.42	*Eremopyrum triticeum*	0.66
Meniocus linifolius	7.72	*Caroxylon nitrarium*	2.01	*Nonea caspica*	0.55
Erodium oxyrhinchum	5.75	*Corispermum lehmannianum*	1.63	*sp3*	0.42
Trigonella arcuata	5.27	*Silene nana*	1.62	*stragalus membranaceus*	0.38
		Arnebia decumbens	1.41	*sp1*	0.37
		Atriplex patens	1.02	*sp2*	0.35
		Carpesium abrotanoides	1.01	*Hypecoum parviflorum*	0.30
				sp6	0.18
				Chrozophora sabulosa	0.15
				Allium mongolicum	0.10
				Ephedra distachya	0.10
				Ferula sibirica	0.09
				sp4	0.09
				Heliotropium ellipticum	0.05
				sp7	0.05
				Echinops sphaerocephalus	0.04
				Isatis tinctoria	0.04
				Erysimum amurense	0.03
				sp5	0.02
				Gagea lutea	0.02
				Amberboa turanica	0.01
				Gypsophila vaccaria	0.01

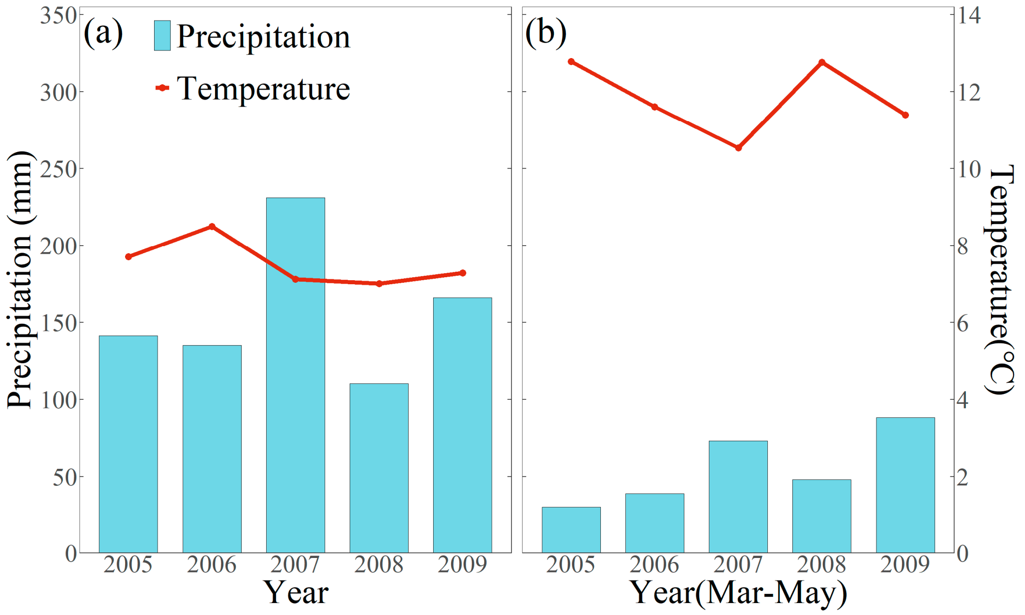

Figure S1. Changes in total precipitation and mean air temperature at the experimental site for the whole year (a) and the growing season (March-May) (b) from 2005 to 2009. Bar charts and line charts represent the trends in precipitation and temperature, respectively.

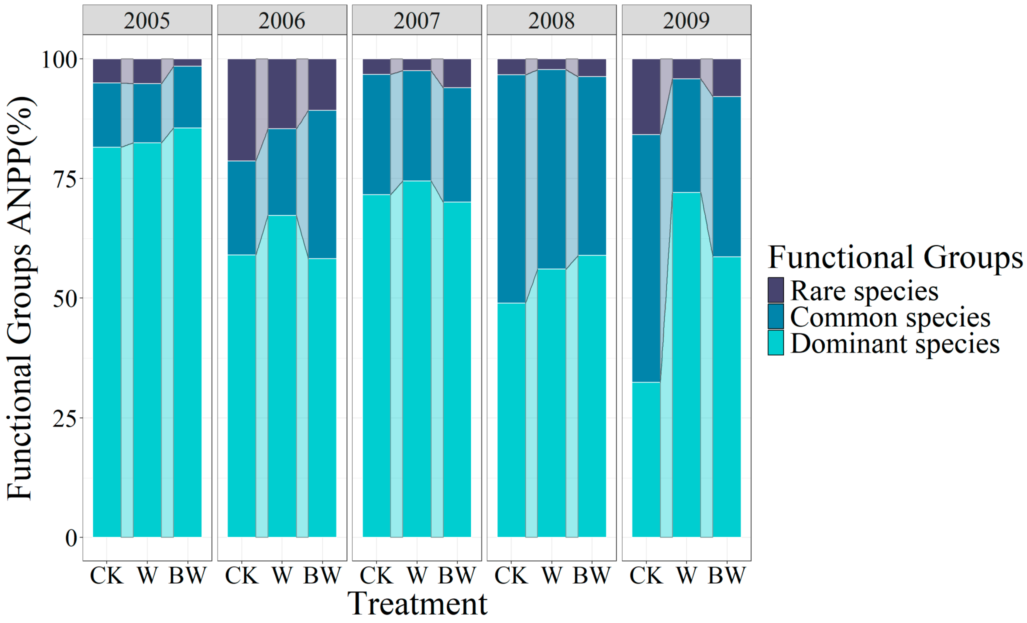

Figure S2. The relative abundance of three functional groups within the plant community based on ANPP under increased precipitation (W) and suppression of AM fungi (BW) during 2005-2009. CK, control; W, only water addition; BW, Benomyl with water.

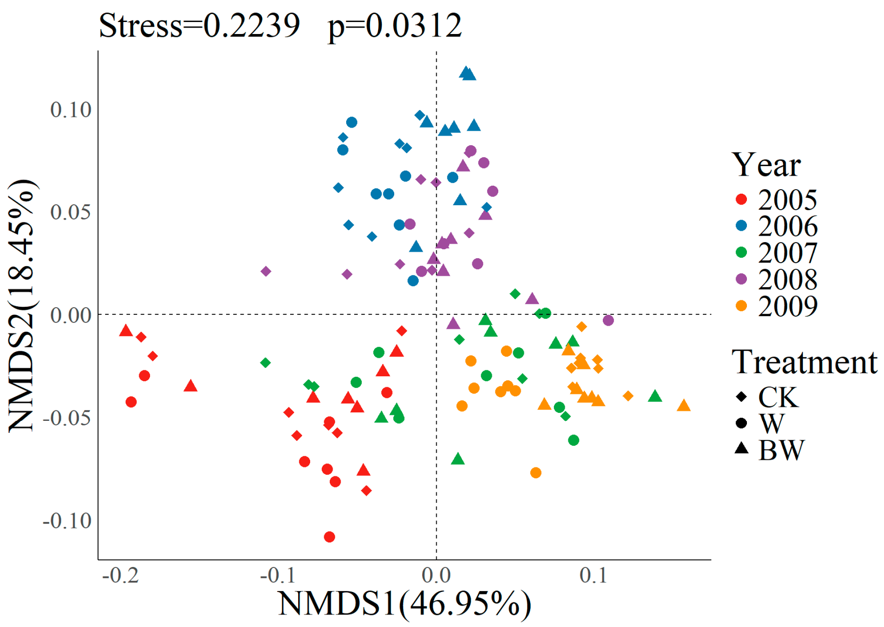

Figure S3. NMDS analysis shows the impact of increased precipitation (W) and suppression of AM fungi (BW) on plant community structure from 2005 to 2009. Different colors represent different years, and different shapes of points represent different treatments.
